# AV-101, a Pro-Drug Antagonist at the NMDA Receptor Glycine Site, Reduces L-Dopa Induced Dyskinesias in MPTP Monkeys

**DOI:** 10.3390/cells11223530

**Published:** 2022-11-08

**Authors:** Mélanie Bourque, Laurent Grégoire, Waseema Patel, David Dickens, Ralph Snodgrass, Thérèse Di Paolo

**Affiliations:** 1 Centre de Recherche du CHU de Québec, Axe Neurosciences, Québec, QC G1V4G2, Canada; 2 Department of Pharmacology and Therapeutics, University of Liverpool, Liverpool L69 3GL, UK; 3 Vistagen Therapeutics, Inc., South San Francisco, CA 94080, USA; 4 Faculté de Pharmacie, Université Laval, Québec, QC G1V0A6, Canada

**Keywords:** L-4-chlorokynurenine, AV-101, MPTP, dyskinesias, NMDA receptors, Parkinson’s disease

## Abstract

N-methyl-D-aspartate (NMDA) receptors have been implicated in L-Dopa-induced dyskinesias (LID) in Parkinson’s disease patients, but the use of antagonists that directly inhibit this receptor is associated with severe side effects. L-4-chlorokynurenine (4-Cl-KYN or AV-101) is a pro-drug of 7-chlorokynurenic acid (7-Cl-KYNA), a potent and specific antagonist of the glycine (GlyB) co-agonist site of NMDA receptors. The 7-Cl-KYNA has limited ability to cross the blood–brain barrier, whereas AV-101 readily accesses the brain. We investigated if AV-101 reduces LID in 1-methyl-4-phenyl-1,2,3,6-tetrahydropyridine (MPTP)-lesioned monkeys while maintaining the antiparkinsonian activity of L-Dopa. A first pilot study using three dyskinetic MPTP monkeys showed that acute AV-101 treatment (250 and 450 mg/kg) reduced LID and maintained the antiparkinsonian activity of L-Dopa. The main study using six additional dyskinetic MPTP monkeys showed that repeated AV-101 treatment (250 mg/kg, b.i.d. for 4 consecutive days) maintained their L-Dopa antiparkinsonian response. We measured significantly less LID when AV-101 was combined with L-Dopa treatment. AV-101 alone or with L-Dopa had no non-motor adverse effects in MPTP monkeys. Our study showed antidyskinetic activity of AV-101 in MPTP monkeys was comparable to amantadine tested previously in our laboratory in this model. We observed no adverse effects with AV-101, which is an improvement over amantadine, with its known side effects.

## 1. Introduction

L-Dopa remains the most effective pharmacotherapy for Parkinson’s disease (PD) patients [1]. However, in a majority of PD patients, chronic L-Dopa treatment is associated with motor complications such as a reduction in the duration of L-Dopa to alleviate motor symptoms (wearing-off) and the occurrence of L-Dopa-induced dyskinesias (LID). LID interfers with quality of life and is challenging to manage [2]. Glutamatergic neurotransmission is increased in the basal ganglia of the brain in PD and LID [3]. Changes in ionotropic and metabotropic glutamate receptors are reported in the brain of parkinsonian patients and parkinsonian monkeys [3]. An approach to diminish LID is through the use of antagonists of ionotropic glutamate N-methyl-D-aspartate receptors (NMDA-R) [3]. In animal models of PD, NMDA-R antagonists reduce LID [3], and some drugs that have NMDA-R antagonistic properties reduce dyskinesias in PD patients [4,5]. However, direct acting NMDA-R channel blocking antagonists have significant side effects limiting their therapeutic utility [6].

NMDA-R can be blocked indirectly at a separate, modulatory site on the receptor, the glycine (GlyB) co-agonist site on the GluN1 subunit [7]. This distinct site is activated by glycine, and GlyB antagonists inhibit glutamatergic neurotransmission. When compared with classical NMDA-R antagonists, GlyB antagonists have a much better safety profile [8]. They do not cause behavioral arousal or motor deficits and do not induce neuropathological or hemodynamic changes [9]. Previous studies have suggested that GlyB antagonists may be useful in treating LID since the GlyB antagonist PAMQX diminished the dose of L-Dopa needed to produce antiparkinsonian therapeutic effects in 1-methyl-4-phenyl-1,2,3,6-tetrahydropyridine (MPTP) monkeys [10]. Further evidence of the utility of GlyB antagonists in treating LID comes from our studies showing that increasing the levels of kynurenic acid, an endogenous GlyB antagonist, in the brains of MPTP monkeys through blockade of kynurenine 3-hydroxylase, reduced LID while maintaining the antiparkinsonian effectiveness of L-Dopa [11,12].

L-4-chlorokynurenine (4-Cl-KYN or AV-101) is a pro-drug of 7-chlorokynurenic acid (7-Cl-KYNA), which is one of the most potent and specific GlyB antagonists known [13]. While AV-101 itself is essentially inactive at the GlyB site [14], 7-Cl-KYNA does not cross the blood–brain barrier, however, the pro-drug, AV-101, which has good pharmacokinetic properties, such as oral availability and an excellent human safety profile, readily gains access to the brain following oral and systemic administration and is efficiently converted to 7-Cl-KYNA [15,16,17]. When injected directly into the brain, 7-Cl-KYNA prevents NMDA-R agonist-induced seizures and neuronal damage [18]. Importantly, AV-101 is preferentially converted to 7-Cl-KYNA and secreted into the neuronal synapses by astrocytes in brain areas that have suffered injury, resulting in a focally enhanced concentration of 7-Cl-KYNA at sites of neuronal injury [19]. These findings indicate that AV-101 has a highly focused site of conversion with local concentrations of newly formed 7-Cl-KYNA greatest at the site of therapeutic need. This would include target brain areas that exhibit hyper-glutamatergic tone [20,21] predicted to be found in the basal ganglia of PD patients. Whether or not AV-101 can be used to decrease LID is currently unknown. Therefore, the present study investigated if the GlyB antagonist AV-101 will decrease LID while maintaining the antiparkinsonian activity of L-Dopa.

## 2. Materials and Methods

### 2.1. In Vitro Uptake of [^3^H]-L-Dopa

L-type amino acid transporter 1 (LAT1, SLC7A5) transport assay was carried out as described previously [22,23,24]. [^3^H]-L-Dopa (specific activity 4 Ci/mmol) was acquired from American Radiolabeled Chemicals, Inc. In brief, HEK293 control cells and HEK293 cells stably expressing LAT1 were plated the day before the experiment. The transporter buffer was comprised of 1 µM L-Dopa (Hellobio, Bristol, UK); 25 mM HEPES (Sigma-Aldrich, Gillingham, UK); pH 7.4; Hank’s buffered saline solution (HBSS) (Sigma-Aldrich, Gillingham, UK); and 0.1% *w*/*v* BSA (Sigma-Aldrich, Gillingham, UK). Individual amino acids used to make up the physiological amino acid solution were purchased from Sigma-Aldrich and reconstituted, as described previously [24]. Cells were incubated with 0.15 µCi/mL [^3^H]-L-Dopa as a radiotracer in the transport buffer with or without AV-101 (1, 2.5, 5 or 10 mM) for 3 min at 37 °C. Cells were washed three times with ice-cold HBSS, lysed with 5% sodium dodecyl sulfate (Sigma-Aldrich, Gillingham, UK) and radiation levels were measured by liquid scintillation counting.

### 2.2. Animals

A pilot study was first performed with three female ovariectomized cynomolgus monkeys (*Macaca fascicularis*) (Charles River Lab, Reno, Nevada, USA) aged 4–6.5 years and weighing 2.74–4.73 kg. Then, the main experiment included six female ovariectomized cynomolgus monkeys (*Macaca fascicularis*) (Charles River Lab, Reno, Nevada, USA) aged 9.2–12.7 years and weighing 3.6–5.2 kg. Handling of the primates was performed in accordance with the National Institute of Health Guide for the Care and Use of Laboratory Animals. All procedures, including the means to minimize discomfort, were reviewed and approved by the Institutional Animal Care Committee of Université Laval. The animals were rendered parkinsonian by continuous infusion of MPTP (Sigma-Aldrich, Oakville, ON, Canada) using subcutaneous Alzet osmotic minipumps (0.5 mg/24 h) until they developed a stable parkinsonian syndrome. Reproducible dyskinesias were induced with the daily oral administration (p.o.) of L-Dopa 100/25 capsules (Prolopa, Hoffmann-La Roche; a mixture of 100 mg of L-Dopa and 25 mg benserazide) for about one month or until dyskinesias stabilized. Prior to this study, the animals had been used in a variety of experiments over many years. An interval of at least one month was left between experiments to allow the experimental drug tested to washout and the animal to rest. During this period, they received L-Dopa at a dose and frequency appropriate to treat their parkinsonian disability.

### 2.3. Drugs

L-4-chlorokynurenine (4-Cl-KYN or AV-101) was provided by VistaGen Therapeutics, Inc. (South San Francisco, CA, USA). AV-101 (250 and 450 mg/kg) was diluted with the vehicle (propylene glycol, 25% in water) and administered by nasogastric gavage (4.5 mL/kg). L-Dopa methyl ester (Sigma-Aldrich, Oakville, ON, Canada) was given subcutaneous (s.c.) at a fixed dose tailored for each animal (11–35 mg/kg), always in combination with benserazide (s.c., 50 mg total) (thereafter called L-Dopa). The tailored dose of L-Dopa was determined to elicit an optimal antiparkinsonian response and clear dyskinesias while limiting side effects such as stereotypies and hypotension.

### 2.4. Pilot Study

**Figure 1:**
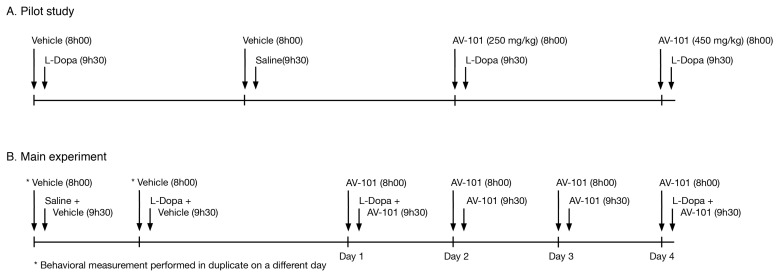
Schematic timeline of AV-101 and L-Dopa administration in monkeys. (**A**) In the pilot study, AV-101 (250 and 450 mg/kg) was administered to 3 monkeys p.o. s.i.d. by nasogastric gavage 1.5 h before L-Dopa. (**B**) In the main experiment, AV-101 (250 mg/kg) was administered to 6 monkeys p.o. b.i.d. for 4 consecutive days in combination with L-Dopa on days 1 and 4, and a behavioral assessment was carried out on days 1, 2, 3 and 4. In both experiments, each monkey was its own control and received the treatments as indicated in the timeline.

A pilot study was conducted on three monkeys and tested two doses (250 and 450 mg/kg) of AV-101 combined with L-Dopa (Figure 1A). AV-101 (250 and 450 mg/kg) was administered p.o. once in combination with L-Dopa s.c. (30–35 mg/kg), and a behavioral assessment was carried out over the duration of the motor effect. L-Dopa was given 1.5 h after AV-101 administration.

### 2.5. Main Experiment

Six monkeys (different from those of the pilot study) were used for the main experiment (Figure 1B). Based on the results of the pilot study, AV-101 (250 mg/kg) was administered p.o. b.i.d. for four consecutive days in combination with L-Dopa s.c. (11–35 mg/kg) on days 1 and 4, and behavioral assessments were carried out on days 1, 2, 3 and 4. On combination days of AV-101 with L-Dopa, L-Dopa s.c. was given first 1.5 h after the first AV-101 administration. The second administration of AV-101 (250 mg/kg) was given 1.5 h after the first one (at the same time as L-Dopa for days 1 and 4).

### 2.6. Behavioral Assessments

Behavioral responses of each animal were video-recorded following dosing with AV-101 with or without L-Dopa. A person other than the observer blinded the video recordings. Thereafter, an observer, blinded to the treatment phase, assessed the motor responses according to the parkinsonian and dyskinetic scales developed at Laval University [25]. Spontaneous locomotor activity was quantified using the Viewpoint electronic monitoring system (VigiePrimates; Viewpoint, Lyon, France).

### 2.7. Data Analysis

For each treatment day and monkey, a mean parkinsonian score and a mean dyskinetic score were obtained by averaging all 15 min scores obtained for the duration of the response. Data from the monkey experiments were analyzed with an analysis of variance (ANOVA) for repeated measures followed by a Dunnett’s multiple comparison test or by a paired *t*-test using GraphPad Prism (version 9.3.1; GraphPad Software, La Jolla, CA, USA). Data from uptake assays were analyzed by one-way ANOVA followed by Tukey’s multiple comparison test. Results are presented as the mean ± S.E.M. A *p*-value ≤ 0.05 was considered significant.

## 3. Results

### 3.1. L-Dopa Transport via LAT1 Is Inhibited Only by High Concentrations of AV-101

**Figure 2:**
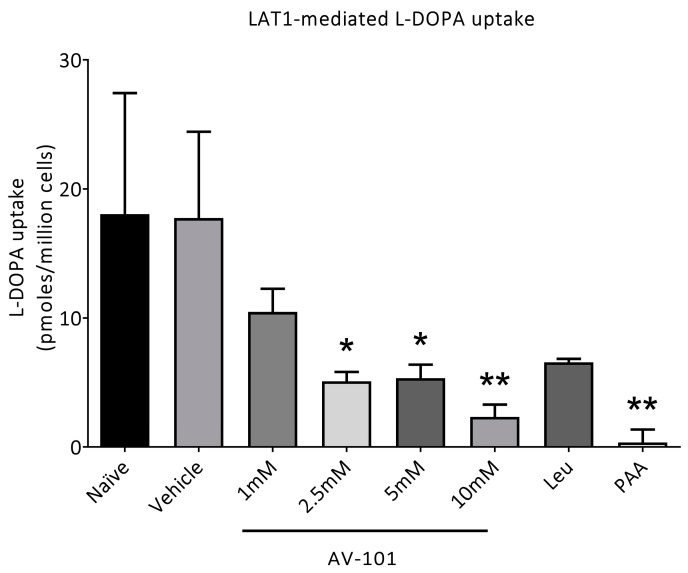
L-Dopa transport via LAT1 is inhibited by high concentrations of AV-101. The uptake of [^3^H]-L-Dopa under conditions indicated. The LAT1-mediated uptake of L-Dopa was determined by subtracting the uptake in HEK 293 control cells from the uptake in HEK 293-LAT1 cells. Cells were exposed to 1µM L-Dopa in the presence of the concentrations of AV-101 indicated for 3 min. Leu (1 mM leucine) and PAA (physiological concentration of plasma amino acids). Data are mean ± S.D. (*n* = 3). * *p* < 0.05 and ** *p* < 0.01 compared to naïve cells. Naïve cells are cells with no drug treatment or vehicle, while the vehicle is phosphate-buffered saline titrated to pH 7.4 using 1 M NaOH.

To investigate the interaction of L-Dopa with AV-101, we carried out an in vitro uptake assay to determine if the LAT1-mediated transport of L-Dopa could be inhibited by a range of AV-101 concentrations (F (7, 15) = 7.103, *p* = 0.0008) (Figure 2). AV-101 at 1 mM did not significantly interfere with the L-Dopa uptake via inhibiting LAT1 transport, while high concentrations reduced the L-Dopa uptake. Leucine at 1 mM did not interfere with the L-Dopa uptake, whereas the physiological concentration of plasma amino acids greatly reduced it in accordance with the known inhibition of the L-Dopa uptake by dietary amino acids [8,26,27].

### 3.2. Acute Treatment with AV-101 and LID (Pilot Study)

**Figure 3:**
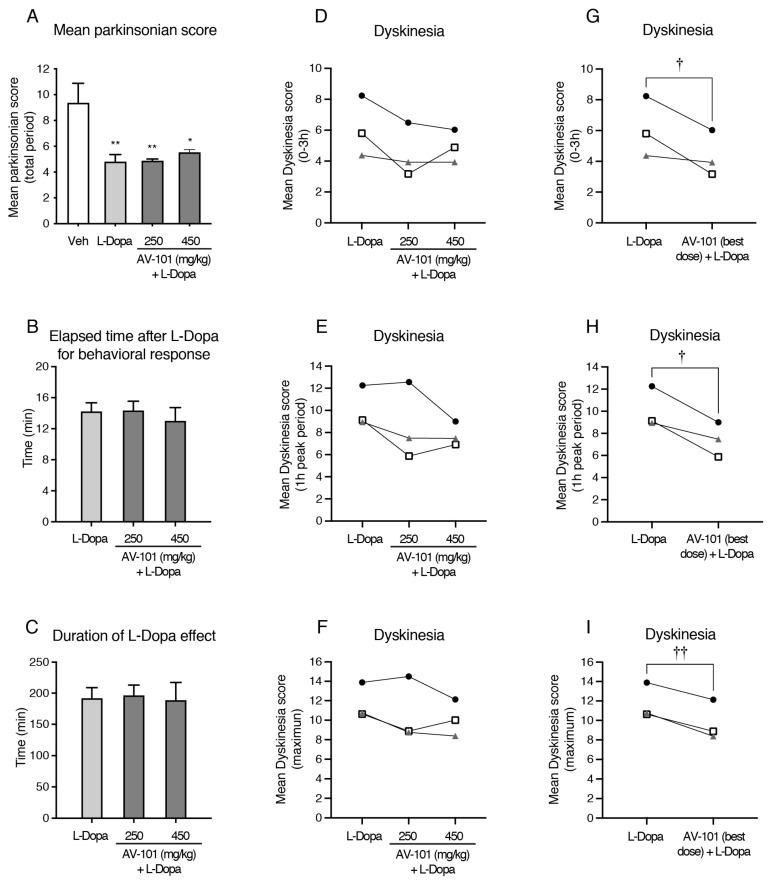
Effect of combination of AV-101 to L-Dopa on the dyskinesia score and antiparkinsonian response in a pilot study (*n* = 3). (**A**) AV-101 maintained the L-Dopa antiparkinsonian response. (**B**,**C**) Delay of L-Dopa response and duration to L-Dopa effect were not affected by the AV-101 treatment. (**D**–**I**) Combination of AV-101 with L-Dopa reduced the mean dyskinesia scores. * *p* < 0.05 and ** *p* < 0.01 vs. Vehicle; † *p* ≤ 0.05 and †† *p* < 0.01 vs. L-Dopa.

L-Dopa improved the parkinsonian score as compared to the vehicle administration (Figure 3A) (F (3,2) = 10.36, *p* = 0.0087). The antiparkinsonian response of L-Dopa was maintained when combined with AV-101. The elapsed time after L-Dopa administration for the start of a behavioral response (thereafter called delay) and duration of the motor response of L-Dopa were not modified by the treatment with AV-101 (Figure 3B,C) (delay: F (2,2) = 1.204, *p* = 0.3897; duration: F (2,2) = 0.2664, *p* = 0.7787). The combination of AV-101 to L-Dopa reduced the mean dyskinesia score by 27–29% (0–3 h and 1 h peak period) (Figure 3D–I) (0–3 h: t(2) = 2.623, *p* = 0.0599; 1 h peak: t(2) = 4.471, *p* = 0.0233; maximum: t(2) = 9.468, *p* = 0.0055).

### 3.3. Repeated Treatment with AV-101 Decreases LID (Main Experiment)

**Figure 4:**
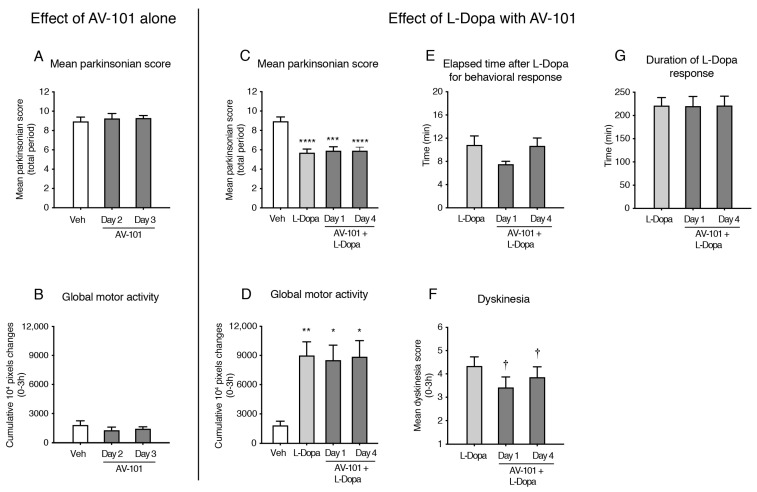
Effect of repeated treatment with AV-101 alone or combined with L-Dopa (main experiment, *n* = 6). (**A**,**B**) AV-101 alone did not change the parkinsonian score of the MPTP monkeys nor did it change their motor activity. (**C**,**D**) L-Dopa improved the parkinsonian score and global motor activity of the animals as compared to the vehicle administration. AV-101 maintained the response of L-Dopa. (**E**,**G**) AV-101 did not delay the motor response of L-Dopa nor did it change the duration of activity. (**F**) AV-101 at 250 mg/kg reduced the mean dyskinesia scores at the 0–3 h period when added to L-Dopa compared to treatment with L-Dopa alone. * *p* < 0.05, ** *p* < 0.01, *** *p* < 0.001 and **** *p* < 0.0001 vs. Vehicle; † *p* ≤ 0.05 vs. L-Dopa.

Figure 4A,B shows the mean parkinsonian score for the total period of the motor effect following the administration of the vehicle compared to the second and third day of the administration of 250 mg/kg of AV-101 alone. AV-101 alone did not change the parkinsonian score (F (2,5) = 1.463, *p* = 0.2771) of the MPTP monkeys nor did it change their motor activity (F (2,5) = 3.256, *p* = 0.0881) (Figure 4A,B). The parkinsonian score of the animals was improved with L-Dopa treatment as compared to vehicle administration; this was maintained when L-Dopa was combined with AV-101 (Figure 4C) (F (3,5) = 184.6, *p* < 0.0001). Higher global motor activity after the administration of L-Dopa alone or L-Dopa combined with AV-101 was measured as compared to the vehicle (Figure 4D) (F (3,5) = 20.71, *p* = 0.0018). The delay and duration of the motor response of L-Dopa were not changed by treatment with AV-101 (Figure 4E,G) (delay: F (2,5) = 4.441, *p* = 0.0431 and post hoc tests not significant; duration: F (2,5) = 0.0281, *p* = 0.9624). Figure 4F shows that AV-101 significantly reduced LID as compared to the L-Dopa alone treatment (F (2,5) = 11.32, *p* = 0.0072).

**Figure 5:**
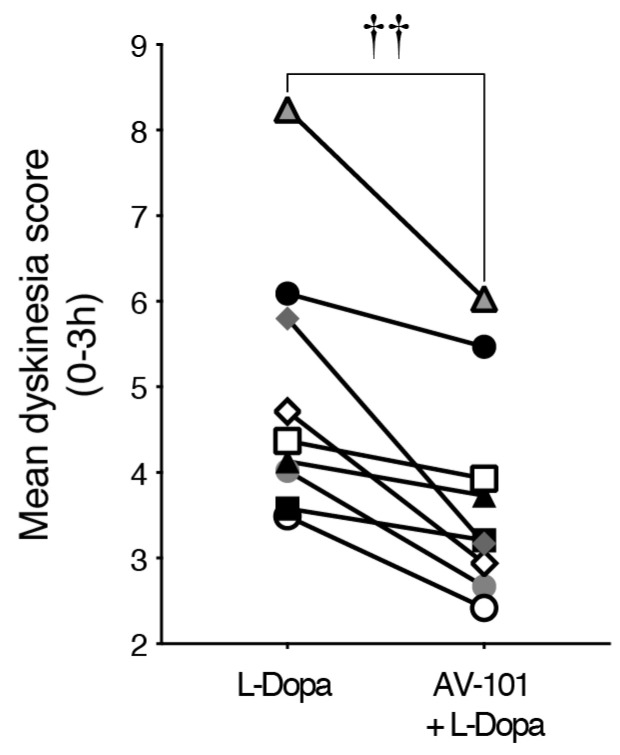
Antidyskinetic effect of AV-101 with L-Dopa in nine different MPTP monkeys (data from the pilot study and the main experiment) showing consistent antidyskinetic activity. †† *p* < 0.01 vs. L-Dopa.

The mean dyskinesia score of the animals tested in both experiments were grouped in Figure 5, showing consistent antidyskinetic activity of AV-101 (t(8) = 4.299, *p* = 0.0029).

## 4. Discussion

The present study showed that administration of AV-101 reduces LID in MPTP-lesioned monkeys, and that AV-101 did not affect the antiparkinsonian response of L-Dopa.

Kynurenic acid and 7-Cl-KYNA have antagonist activity at the GlyB co-agonist site of the NMDA-R; however, 7-Cl-KYNA is approximately 20-fold more potent at this site than endogenous kynurenic acid [13]. We previously showed that increasing kynurenic acid levels with the kynurenine 3-hydroxylase inhibitor Ro 61-8048 inhibited the development of LID in de novo MPTP monkeys [11]. In MPTP monkeys with LID, acute Ro 61-8048 administration reduces the dyskinetic response to L-Dopa by about 20% while having no effect when administered alone, on the parkinsonian score [12]. In the present study, AV-101 reduced LID by about 25% and maintained the L-Dopa antiparkinsonian activity further supporting the GlyB co-agonist site of NMDA-R as a novel target to reduce LID.

A similar reduction in LID, as with AV-101, is also reported with amantadine, another NMDA-R antagonist and the only drug considered clinically useful for the management of LID [28]. For comparison, amantadine treatment reduces by about 25% dyskinesias in our MPTP monkeys [29,30], however, this is associated with a reduction in the antiparkinsonian effect of L-Dopa. Furthermore, amantadine is known to induce adverse side effects (in humans and in MPTP monkeys) [28]. We observed no adverse effects with AV-101 treatment in our monkeys, as also reported in humans in a clinical phase 1 study showing that AV-101 was safe and well tolerated [17]. The United States Food and Drug Administration approved a phase 2 clinical trial to evaluate the efficacy and safety of AV-101 in PD patients with LID (ClinicalTrials.gov identifier: NCT04147949).

Both L-Dopa and AV-101 are transported across the blood–brain barrier by LAT1. AV-101 was administered 1.5 h before L-Dopa treatment (in both experiments) and at the same time as L-Dopa in the main experiment. AV-101 rapidly gains access to the brain. Although the second administration of AV-101 was given at the same time as L-Dopa in the main experiment, this did not modify the L-Dopa antiparkinsonian response. The delay and duration of the motor response as well as the parkinsonian score after L-Dopa administration were not changed by AV-101 treatment, suggesting that AV-101 did not affect the access to the brain of L-Dopa. This is consistent with our in vitro results (Figure 2) showing that substantial concentrations of AV-101 (≥2.5 mM) were needed to inhibit the LAT1-mediated L-Dopa uptake. As pharmacokinetic studies in humans following the oral administration of 1440 mg AV-101 find a plasma concentration of 250µM [17], it seems unlikely that AV-101 will interfere with the transport of L-Dopa by LAT1.

AV-101 has a short half-life of about 1.73 h, as reported in humans [17]. For this reason, in the main experiment, AV-101 was administered 1.5 h before and at the same time of L-Dopa to extend the duration of the effect of AV-101. A recent study also reported that 7-Cl-KYNA, which binds to the GlyB co-agonist site of NMDA-R, leaves the brain extracellular fluid via probenecid-sensitive organic anion transporters [24]. Indeed, the co-administration of probenecid with AV-101 increased the brain concentration of 7-Cl-KYNA [24]. Whether the co-administration of AV-101 and probenecid could have a beneficial effect on LID remains to be investigated.

The pro-drug nature of AV-101, i.e., the enhanced delivery of the active drug in a focused way to the relevant regions of the brain undergoing pathological stress, reduces the possibility of systemic side effects with long-term use. AV-101 is taken up into the brain by the LAT1 transporter at the blood–brain barrier and is preferentially converted to 7-Cl-KYNA by astrocytes [15]. AV-101 was reported to have a highly focused site of conversion; a higher 7-Cl-KYNA level subsequent to 4-Cl-KYN treatment was found in brain areas involved in seizure initiation following the administration of kainate [19]. Thus, this suggests that local concentrations of newly formed 7-Cl-KYNA are greatest at the site of therapeutic need and target brain areas that exhibit glutamatergic overactivity, as in the basal ganglia of PD patients. This provides an advantage over direct acting GlyB antagonists which block NMDA-R channels throughout the body, or inhibitors of kynurenine 3-hydroxylase that will raise kynurenic acid levels globally in the brain, thereby making AV-101 a unique candidate for the management of LID in PD patients.

This study used the MPTP monkey, a well-established neurotoxic model of PD that is pharmacologically validated for the assessment of parkinsonism and dyskinesia [31,32]. Hence, the present results are limited to the context of advanced PD since severe nigrostriatal denervation and drug sensitization are modeled [33,34]. Another limitation of the present study was the acute and sub-chronic investigation of AV-101. Future experiments should test if the antidyskinetic activity of AV-101, with or without probenecid, is maintained in the long-term, since, in humans, long-term use is the likely application. Since, we have used L-Dopa s.c., it would be informative to test oral administration, the common mode of administration in humans, in MPTP monkeys of both sexes.

In conclusion, our results show that the pro-drug AV-101 decreases LID while maintaining the antiparkinsonian response of L-Dopa. This provides support that targeting the GlyB co-agonist site of NMDA-R could be a useful approach for the management of L-Dopa-induced motor complications in PD.

## Data Availability

The data presented in this study are available upon reasonable request from the corresponding author.
